# Toward a Better Understanding of Emotional Exhaustion Among First-Line Nurse Managers: The Role of Psychological Stress, Presenteeism, and Leader Loyalty

**DOI:** 10.1155/jonm/8865098

**Published:** 2025-12-01

**Authors:** Véronique Achmet, Martin Lauzier

**Affiliations:** ^1^Department of Administrative and Business Management, University of Bordeaux, Institut de recherche en gestion des organisations (IRGO), Bordeaux, France; ^2^Industrial Relations Department, Université du Québec en Outaouais, Gatineau, Canada

**Keywords:** emotional exhaustion, leader loyalty, mediated moderation, presenteeism, psychological stress

## Abstract

**Aims:**

Based on the Conservation of Resources and Leader–Member Exchange theories, this study investigates the relationship between psychological stress and emotional exhaustion among first-line nurse managers (FLNMs), while also examining the mediating role of presenteeism (i.e., working while ill) and the moderating effect of leader loyalty on this relationship.

**Method:**

A survey-based study was carried out on 161 French FLNMs. A mediated moderation model was tested through bootstrap regression analyses using PROCESS Macro.

**Results:**

The analyses yielded three primary findings. First, psychological stress among FLNMs is positively related to their level of emotional exhaustion. Second, presenteeism is found to be an explanatory mechanism of the psychological stress—emotional exhaustion relationship. Third, leader loyalty was found to moderate the indirect link between psychological stress (i.e., through presenteeism) and emotional exhaustion.

**Conclusion:**

The results provide a better understanding of the relationship between psychological stress and emotional exhaustion in the case of French FLNMs. They also clarify the role of other mechanisms, such as presenteeism and leader loyalty, that may be involved.

**Practical Implications:**

Considering the pivotal role of FLNMs within hospitals, findings from this study raise awareness of the detrimental consequences of psychological stress and presenteeism on FLNMs' health, while also showing the constructive aspects of the relationships between FLNMs and their superiors.

## 1. Introduction

As significant stakeholders in healthcare settings, first-line nurse managers (FLNMs) play a pivotal role [1] by overseeing the largest workgroup in the healthcare system, i.e., nurses [2]. FLNMs' role is central in managing their organization's core values and clarifying organizational orientations to staff, while also encouraging continuous adherence to professional/ethical standards [2]. FLNMs are also tasked with motivating nurses to achieve their professional objectives and create work environments conducive to patient care [3]. It is extremely important that FLNMs identify and implement strategies to enhance job satisfaction among team members so that staff turnover is reduced [4]. Faced with daily work demands and pressures that can affect both their health and well-being, FLNMs have been recognized as occupying the most stressful occupation in healthcare [3, 5]. As a result, FLNMs are prone to both psychological stress [1] and emotional exhaustion [6].

The main objective of this study is to investigate the relationship between psychological stress and emotional exhaustion among FLNMs. Psychological stress concerns “a particular relationship between the person and the environment that is appraised by the person as taxing or exceeding his or her resources and endangering his or her well-being” ([7]; p. 19). Long-term stress at work without sufficient recovery is related to higher exhaustion rates and can seriously impede workers' health, morale, and performance [1, 8]. Emotional exhaustion, which refers to “the feeling of being emotionally overextended and depleted of one's emotional resources” ([9], p. 69), has been shown to have organizational consequences, including increased quitting intentions [10, 11]. While the effects of this kind of extreme fatigue among nurses have been abundantly characterized (e.g., [12, 13]), very few studies have focused on FLNMs as a distinct group. This study aims to gain a better understanding of the consequences of psychological stress on FLNMs' emotional exhaustion and the underlying mechanisms that might influence this state. Due to its potential for limiting the recovery process [12, 14, 15], as complementary objectives, this study also aims to highlight the influence of presenteeism as a mediating mechanism and leader loyalty as a moderating effect in the connection between psychological stress and emotional exhaustion.

This study contributes to the existing body of knowledge in several ways. First, it examines emotional exhaustion in a working population that has hitherto received little attention (see [8]). Although FLNMs are often included in larger worker samples, very few studies have considered them as the main subject of investigation. Second, this study analyzes the consequences of psychological stress on FLNMs' emotional exhaustion, emphasizing the mediating role of presenteeism and the moderating role of leader loyalty. In accordance with the *Conservation of Resources* (COR) *Theory*, this study first explores how psychological stress directly impacts FLNMs' emotional exhaustion. It then examines the role of presenteeism as an explanatory mechanism for the relationship between psychological stress and emotional exhaustion. Lastly, based on *Leader–Member Exchange* (LMX) *Theory*, the study examines leader loyalty's moderating (i.e., buffering) role. According to LMX theory, leader loyalty refers to the expression of public support by one member (e.g., the superior) for another member (e.g., the subordinate). This research investigates the extent to which FLNMs' perceived loyalty toward their superiors moderates the relationship between presenteeism and emotional exhaustion.

## 2. Theoretical Background

### 2.1. Linking Psychological Stress to Emotional Exhaustion

Due to the complexity of their role, FLNMs face many demands that can affect both their health and well-being [8]. This can be explained, in part, by the fact that FLNMs fulfill many crucial tasks in healthcare settings given their primary responsibility for such roles as coordinating and monitoring the quality of patient care, and assessing staff performance. FLNMs also facilitate communication, strengthen professional relationships, and develop and support the overall functioning of work teams [16]. Hence, the cumulative effect of these tasks and the pace at which they are to be achieved can sometimes contribute to exacerbating FLNMs' stress levels [5]. The level of stress experienced can also be increased by the additional challenges that FLNMs face: the scarcity of resources, staffing patterns, managing staff expectations, and leadership skills [17, 18]. To illustrate, FLNMs are responsible for managing daily schedules and coordinating the replacement of nurses, often with short notice, to ensure continuity of care. In a context of budget cuts, this task could generate very high levels of stress for FLNMs [19]. In a similar vein, Warshawsky et al. [20] found that FLNMs reported budget constraints to be incompatible with providing the best and safest patient care, a situation they described as ‘draining.'

In view of the professional environment within which they operate, FLNMs experience psychological stress on a daily basis, which most often results in forgetfulness and poor concentration [1]. The exhausting character of FLNMs' work [5] makes them a vulnerable group at risk for ill health and reduced psychological well-being [8]. Indeed, this factor may have serious consequences for not only FLNMs' own health but also the quality of care they provide [8]. The present study draws on the COR theory, as did those of Luo et al. [21] and Zhang et al. [22], who have also worked on this population. According to the COR theory, emotional exhaustion is most likely to occur in three scenarios: (a) actual resource loss, (b) a perceived threat of resource loss leading to inadequate levels for meeting work demands, and (c) inadequate resource gains obtained from investments in resources [23]. Like nurses, FLNMs are exposed to heavy workloads, low rewards, and poor social support [24]. Considering the challenges mentioned previously (see also [17, 18]), FLNMs' tasks make them prone to emotional exhaustion [6], which can be seen as the final stage of prolonged and extensive exposure to stressful conditions [25]. This feeling is further intensified when workers perceive that their investment of resources does not yield the desired returns [26]. This can have a detrimental effect on their health [27]. Some studies have indicated that the psychosocial resources and networking opportunities provided to FLNMs were insufficient to mitigate their workplace stress [28]. Moreover, other research has highlighted that a paucity of support from department heads was a prominent factor contributing to FLNMs' decision to resign from their positions [29]. Given these scenarios, it seems reasonable to posit that FLNMs' perceived stress levels will correlate positively with their emotional exhaustion.

  H1. Psychological stress correlates positively with emotional exhaustion.

### 2.2. Mediating Role of Presenteeism

Notwithstanding, previous research on emotional exhaustion has noted that there are still unanswered questions regarding its processes [30]. Some authors have suggested that presenteeism (i.e., working while ill) may be an underlying mechanism that can help better understand this state of fatigue. This idea draws on the work of Nicolas et al. [31], who emphasize the importance of presenteeism by refocusing on the reasons that might motivate such a way of behaving, i.e., when under a compromised state of health. While several studies focus primarily on presenteeism among nurses [13, 32, 33], to the best of the authors' knowledge, there is a paucity of research examining the phenomenon of presenteeism among FLNMs.

It has been established that workplace stress is linked to higher levels of presenteeism [34] and can negatively impact the ability of workers to concentrate or perform their work normally despite impaired health [35]. Indeed, it is reasonable to assume that presenteeism-associated stress can contribute to a decline in work performance (e.g., higher rate of errors, accidents, and/or injuries) and mental functions (e.g., impaired attention and memory lapses). Such impacts are often due to a combination of stress and fatigue in workers [14, 36], both of which are linked to presenteeism [12, 37]. In addition, given that FLNMs must meet demanding work requirements [1] that increase the likelihood they will come to work when ill [38], these demands can trigger presenteeism via the impaired health path, resulting in elevated state of fatigue and more medical problems [39, 40]. To avoid potential resource loss due to increased job demands, employees may draw on other available resources, including continuing to work while sick [12]. While people can cope with stressful conditions and develop different approaches to dealing with the source of the stress (e.g., confronting it), some workers, despite impaired health, may feel they have to come to work for other reasons (e.g., to avoid disappointing their superiors and/or colleagues) [41].

Likewise, other findings based on a sample of nurses indicate that presenteeism can be associated with emotional exhaustion [12]. Indeed, due to its harmful effects on the recovery process [14, 15], presenteeism is likely to lead to higher levels of emotional exhaustion [13, 42]. Previous research has shown that healthcare professionals working in a suboptimal state of health are more prone to mistakes [43]. Research to date has also shown that presenteeism is linked to higher levels of stress [34] and increased fatigue [37]. Based on these ideas, this study posits presenteeism as a mediating mechanism of the relationship between psychological stress and emotional exhaustion in the case of FLNMs.

  H2. Presenteeism plays a mediating role in the relationship between psychological stress and emotional exhaustion.

### 2.3. Moderating Role of Leader Loyalty

In this study, the concept of leader loyalty is considered as a facet of the LMX [44]. Leader loyalty refers to the expression of public support by one member (e.g., the superior) for the goals, character of another member [44, 45]. Graen and Uhl-Bien [46] posit that the most effective demonstration of leader loyalty occurs in a “mature partnership” wherein superiors and subordinates are cognizant of their reciprocal dependence. The perception of a high level of trust, respect, and mutual obligations indicates that subordinates rely on their superior for the support and encouragement they need [45]. In the present study, the perspective of the subordinates (FLNMs) was considered with the objective of gaining insight into how they perceive the leader loyalty of their superiors (i.e., middle nurse managers). Contrary to the extant research literature, the importance of LMX (which is sourced at different hierarchical levels) in relation to employees' work experience remains understudied [47]. The majority of studies in this field have focused on the overall concept of leadership rather than examining the specific nuances of LMX relationships across different hierarchical levels. In line with Dionne's [48] research, this study aims to examine perceptions of leader loyalty.

Considering the beneficial effects demonstrated in previous studies, leader loyalty appears to be a crucial factor in job satisfaction [48] and innovative behaviors [49]. In line with the LMX theory, Lai et al. [50] suggest that positive exchanges between leaders and members represent a valuable resource that can facilitate subordinates' ability to cope more effectively with their work challenges. Although supportive relationships with superiors can help mitigate subordinates' burnout, especially emotional exhaustion [51], there is a paucity of research on the relationships between FLNMs and their superiors. This study, therefore, examines the moderating role of leader loyalty on the relationship between psychological stress and emotional exhaustion.

  H3. Perceived leader loyalty moderates the relationship between presenteeism and emotional exhaustion by weakening the relationship when perceived leader loyalty increases and vice versa.

### 2.4. A Model of Mediated Moderation (MEDMOD)

**Figure 1:**
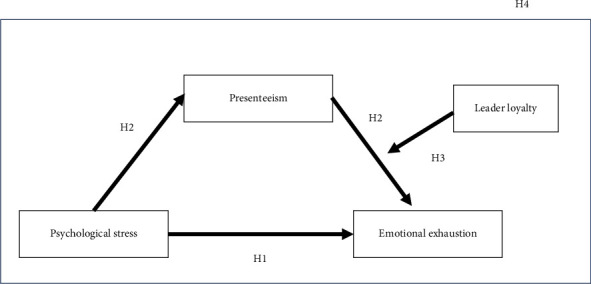
The proposed mediated moderation model.

The preceding arguments suggest a hypothesis of a MEDMOD model (see Figure 1) to the effect that psychological stress initially affects presenteeism, which in turn affects emotional exhaustion. This model also suggests that the effect of psychological stress on emotional exhaustion (through presenteeism) is a function of leader loyalty from FLNMs' standpoint. To the best of the authors' knowledge, this study appears to be the first to integrate these two effects within the same model, thereby offering a more nuanced lecture of these relationships.

  H4. The indirect effect of psychological stress on emotional exhaustion (through presenteeism) depends on the level of leader loyalty, with the result that this relationship becomes weaker as perceived leader loyalty increases and vice versa.

## 3. Methods

### 3.1. Study Context and Data Collection

This cross-sectional study was conducted in the French healthcare sector, which has undergone a series of reforms over recent decades [19]. These successive waves of reform have significantly altered French healthcare workers' employment context and working conditions. In the face of the cumulative impact of these reforms, FLNMs are compelled to implement new management practices, address the strategic challenges of regional care organization schemes, analyze and monitor their departments' costs, and monitor/maintain standards of professional excellence [52].

Prior to the administration of the questionnaire, a pretest was conducted. A group of nine FLNMs operating within the French public hospital sector was invited to identify any potential misunderstandings. It was observed that there were no reported misunderstandings regarding the questionnaire, which facilitates its dissemination.

Participation in this study was voluntary. Participants were instructed to access Eval&Go software to complete an electronic survey comprising validated versions (in French) of scales for all studied variables. This software is compliant with General Data Protection Regulation (GDPR). In conformity with our university rules and regulations concerning the solicitation of human subjects for research purposes, this study's objectives and the participants' rights were clearly stated on the first page of the survey. Participants' consent was recognized by survey participation (i.e., by filling in the survey), and data collection was considered confidential and anonymous as it could not be linked back to a specific participant. At the time of the survey, a decision by the research ethics committee was not required. However, approval has been received from the hospital authorities. Subsequently, the healthcare management teams distributed the link to the online questionnaire to FLNMs.

### 3.2. Participants

This study sample includes 161 French FLNMs, 133 (83%) of whom were female. While most respondents were aged between 35 and 49 years, their ages broke down as follows: 1 participant between 25 and 29 years (i.e., 0.6%), 15 between 30 and 34 years (i.e., 9.3%), 35 between 35 and 39 years (i.e., 21.8%), 31 between 40 and 44 years (19.2%), 36 between 45 and 49 years (22.3%), 24 between 50 and 54 years (15%), and 19 between 55 and 59 years (11.8%). Most participants worked in healthcare centers (94; 58.4%), while 56 (34.8%) worked in university or regional hospitals. Finally, 11 (6.8%) worked in specialized (psychiatric) hospitals. Ten respondents (6.2%) had less than a year's FLNM experience. The majority of the FLNMs (107; 66.5%) had between 1 and 9 years of experience in their current position, with 39 (24.2%) having between 10 and 19 years, and only 5 (3.1%) having between 20 and 29 years.

### 3.3. Measures

#### 3.3.1. Emotional Exhaustion

This variable was measured with a French single item (e.g., “*Do you ever feel emotionally exhausted?*”) used by Estryn-Behar et al. [53]. Past studies that have used a similar item to measure emotional exhaustion have reported satisfactory definitional correspondence and moderate reliability (e.g., [54–56]). Then, through the single item, participants had to indicate their degree of exhaustion according to a 5-point scale (1 = *Never*/*almost never*; 5 = *Always*). A high score on this item indicates a higher level of emotional exhaustion.

#### 3.3.2. Psychological Stress

The MSP-9 measure of psychological stress, developed in French by Lemyre and Tessier [57], was used to assess psychological stress. This instrument comprises nine items arranged on a 5-point Likert scale (1 = *Strongly disagree*; 5 = *Strongly agree*). Respondents were asked to indicate the degree to which they had recently experienced stress, i.e., in the previous 4-5 days (e.g., *I felt stressed*). A high score on this measure corresponds to a high level of perceived stress (*α* = 0.77).

#### 3.3.3. Presenteeism

The SPS-6 Stanford Presenteeism Scale, developed in English by Koopman et al. [35], was used to measure presenteeism. This scale consists of six items (e.g., *Despite [my health problem], I have been able to perform the more complex tasks my job includes*). This metric is commonly used in studies on presenteeism and has been validated into French [58]. Participants were asked to respond to these items using a 5-point Likert scale (1 = *Strongly disagree*; 5 = *Strongly agree*). A high score on this measure signifies a greater ability to focus on work and avoid distractions that might arise from a health problem (*α* = 0.62).

#### 3.3.4. Perceived Leader Loyalty

This variable was measured using one dimension of the LMX-MDM instrument developed by Liden and Maslyn [44], adapted into French by El Akremi et al. [59] and used by Robert and Vandenberghe [60]. In this case, the dimension consisted of three items (e.g., *My line manager would defend me to other members of the organization if I made an honest mistake*). Participants were asked to respond to these items using a 5-point Likert scale (1 = *Strongly disagree*; 5 = *Strongly agree*). A high score on this measure corresponds to a high level of perceived leader loyalty from the superior to the subordinate (*α* = 0.90).

#### 3.3.5. Control Variables

Given that previous studies found higher rates of presenteeism among women than men [61], and given also higher prevalence of presenteeism among older workers [34], these variables were controlled in subsequent analyses.

## 4. Results

### 4.1. Preliminary Analysis

In compliance with certain statistical prerequisites, SPSS Version 29 was used to conduct a series of tests on the collected data. Initially, variable distribution indices (skewness and kurtosis) were inspected. As all values fell within accepted thresholds, the variables were deemed sufficiently normal for further analysis. Also, the reliability coefficients (Cronbach's alpha) indicated acceptable values (i.e., over 0.70), albeit a lower alpha for SPS-6 (i.e., the presenteeism measure), thereby validating the use of an overall score for each variable. It is noteworthy that similar presenteeism values have also been observed in previous studies using SPS-6 (e.g., [58, 62]). As shown in Table 1, the correlation between studied variables shows concordant values (both in terms of directionality and magnitudes) with previous studies. Specifically, psychological stress positively correlates with presenteeism (*r* = 0.344, *p* < 0.01) and emotional exhaustion (*r* = 0.213, *p* < 0.01). These few observations are consistent with results reported in past studies [12, 13, 37, 42] and offer initial support for the hypotheses formulated. Finally, it also seems fair to note that loyalty coming from the leader correlates negatively (*r* = −0.161, *p* < 0.05) with emotional exhaustion.

Given the cross-sectional nature of this study, all the collected data were tested to ascertain the presence of common variance bias [63, 64]. According to this test, a strong influence of common variance bias can be considered when a single factor emerges from this analysis and/or when the variance explained by the first factor of the solution is greater than 50% [64]. In this case, the results indicate the presence of multiple factors, with the first factor accounting for only 32% of the total variance, which suggests that common method bias influence (if present) is of a comparable level with that found in other studies using a similar design. Moreover, an evaluation of the variance inflation factors (VIFs) calculated from the multiple regression models revealed that they were of low amplitude (i.e., between 1.04 and 1.15), thus well below the threshold of 4.0 suggested by Hair et al. [65].

Confirmatory factor analyses (CFAs) were also conducted to evaluate both the general structure of the questionnaires and the functioning of each scale. Mplus 8.10 software was used to perform these analyses (Table 2). Assessment of model fit was based on the use of standard goodness-of-fit indices, with interpretation guided by commonly accepted threshold values [66]. These included the *Comparative fit index* (CFI; ≥ 0.95 for excellent, ≥ 0.90 for good), the *Tucker–Lewis index* (TLI; ≥ 0.95 for excellent, ≥ 0.90 for good), and the *root mean square error of approximation* (RMSEA; ≤ 0.06 for excellent, ≤ 0.08 for good) with its 90% CI and the *standardized root mean square residual* (SRMR; ≤ 0.06 for excellent, ≤ 0.08 for good). The indices obtained reveal a satisfactory fit for the three-factor model (*χ*^2^ = 40.407; dl = 24; *χ*^2^/dL = 1.68; CFI = 0.968; TLI = 0.952; RMSEA = 0.065; SRMR = 0.052). As shown in Table 2, these results are superior to those of the one-factor model (*χ*^2^ = 203.434; dl = 27; *χ*^2^/dL = 7.535; CFI = 0.655; TLI = 0.540; RMSEA = 0.201; SRMR = 0.152) and other plausible competing models.

### 4.2. Hypothesis Testing

The various hypotheses were tested through bootstrap regression analyses using the PROCESS Macro (Model 14). As detailed in Table 3, this analysis is based on 5000 replications with corrected and accelerated biases, while controlling for the participants' gender and age.

The first hypothesis (H1) posited a positive relationship between psychological stress and emotional exhaustion. As indicated in Table 3, the direct effect of psychological stress on emotional exhaustion is positive and significant (*β* = 0.19; *ρ* < 0.05).

The second hypothesis (H2) posited that presenteeism serves as a mediating factor in the relationship between psychological stress and emotional exhaustion. There is a positive association between psychological stress and presenteeism (*β* = 0.39; *ρ* < 0.001). Presenteeism was also positively associated with emotional exhaustion (*β* = 0.62; *ρ* < 0.01). The results appearing in Table 3 show a mediating effect on presenteeism. As shown, the strength of the association between psychological stress and emotional exhaustion was approximately halved following the incorporation of presenteeism into the regression model. Since the direct relationship between psychological stress and emotional exhaustion remains significant (i.e., after considering the effect of presenteeism), these observations suggest the presence of a partial mediation effect. These results therefore support H2.

**Figure 2:**
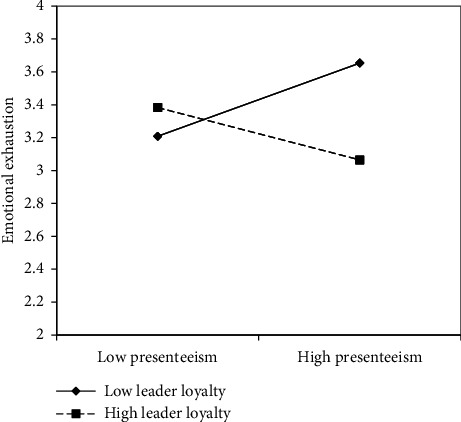
Moderating effect of leader's loyalty.

The third hypothesis (H3) posits that leader loyalty plays a moderating role in the connection between presenteeism and emotional exhaustion, thus suggesting that this connection would become weaker as (perceived) leader loyalty increases and vice versa. As shown in the lower part of Table 3, the results indicate the presence of a significant interactive term (i.e., presenteeism × leader loyalty), albeit with a negative sign (*β* = −0.17; *ρ* < 0.01). This interaction accounts for an additional 4% of the variance in the model. As shown in Figure 2, and in line with the proposed hypothesis, the effect of leader loyalty is most beneficial for FLNMs with higher presenteeism scores. In other words, the loyalty perceived by FLNMs from their superior helps mitigate the effect of presenteeism on emotional exhaustion, particularly among those who more frequently work while sick. As shown in Table 4, this effect appears to be limited to a small proportion of the sample (i.e., the area below the 16th percentile only). Overall, these observations support H3.

Finally, the fourth hypothesis (H4) posits that the indirect effect of psychological stress on emotional exhaustion (through presenteeism) is contingent on the level of leader loyalty. As shown in the lower section of Table 4, the *Index of* MEDMOD is statistically significant (−0.0674; 95% CI = [−0.1471—−0.0076]). These results support H4.

## 5. Discussion

### 5.1. Main Observations

Observations made by this study advance knowledge about psychological stress and provide a more nuanced comprehension of its effect on FLNMs' well-being (i.e., emotional exhaustion). This research adds to recent work on emotional exhaustion among FLNMs (e.g., [21, 67, 68]). Indeed, it highlights presenteeism as a mechanism that explains the effect of psychological stress on levels of emotional exhaustion. This finding provides a more precise picture of the process that can explain the poorer health of FLNMs, who inherently play a pivotal role in hospital settings [1, 2]. This poor health can have implications not only for the teams' FLNM management but also for the patients they serve. The existing literature attests to the high work demands placed on FLNMs [1, 5, 20]. As previously stated, the study is based on the COR theory, which suggests that individuals strive to acquire, protect, and maximize their resources in order to reduce psychological distress and prevent burnout [69, 70]. It posits that stress occurs when an individual's essential resources are depleted, threatened by loss or when an individual fails to obtain the critical resources following significant effort [69]. Resources are defined “as those objects, personal characteristics, conditions, or energies that are valued by the individual or that serve as a means for attainment of these objects” ([69], p. 516). As Warskawsky et al. [20] have found, FLNMs operate within a highly challenging work environment, which has been shown to result in significantly elevated stress levels. Our study's findings indicate that FLNMs may be forced to cope with these demands by drawing on their personal resources in order to turn up for work even when their health requires them to take time off. FLNMs could also develop the belief that they need to turn up for the sake of both patients and subordinates [71]. In other words, they mobilize and often deplete their personal resources to dedicate themselves to the service of their teams and patients.

These circumstances give rise to a phenomenon known as presenteeism (i.e., working while ill), which, unlike for the overall nursing population [12, 32, 72], has been relatively understudied for FLNMs. This study's findings highlight the role of presenteeism by refocusing on its mainspring, a poorer state of health [31]. Presenteeism merits attention due to its adverse impact on work performance [12, 34]. In view of this study's findings, it is possible to envision the manifestation of presenteeism (as measured by SPS-6) in different ways, including reduced vigilance, momentary lapses, and tendencies to shorten certain work procedures. Collectively, these conditions could contribute to increased levels of fatigue and the number of errors, which could ultimately lead to negative impacts on the health of FLNMs and those they serve. The results indicate that this phenomenon also affects this group of hospital workers and reduces their resources, which, in turn, leads to emotional exhaustion. It is imperative to consider the phenomenon of presenteeism as one that could result in long-term adverse consequences for both FLNMs and their organization. These findings raise significant concerns insofar as individuals experiencing higher levels of emotional exhaustion are at an increased risk of developing psychological and physical health issues [73], which can ultimately lead to prolonged work absences [74, 75].

Emotional exhaustion is a core component of burnout [76]. Although burnout has been discussed in the nurse population, FLNMs' burnout has yet to be fully understood [21]. Organizations must give due consideration to the issue for many reasons. First, the development of cynicism and inefficacy are well known consequences of emotional exhaustion [77]. Second, emotional exhaustion occurs when individuals perceive that they lack the necessary resources to perform the tasks required of them and feel emotionally overextended and drained [9]. This being said, provision of support (as conceived through a COR lens) can help individuals coping with prolonged and new demands [78, 79]. This study is also original in that it investigates the moderating role of leader loyalty. The results of this study align with those of Dionne [48], who underscores the importance of understanding leader loyalty by examining the vertical dimension of hierarchical relationships between actors (i.e., FLNMs and their superiors). Beyond this alignment, our findings contribute to addressing a gap in the literature, as the relationship between FLNMs and their superiors remains insufficiently explored. This relationship is particularly relevant in light of the significant changes that have reshaped the French hospital sector following recent public management reforms [80].

From the perspective of LMX relationships, our results suggest that low levels of perceived leader loyalty exacerbate the relationship between psychological stress and emotional exhaustion. In contrast, high levels of loyalty mitigate this effect. These findings highlight the pivotal role of hierarchical relationships in contributing to emotional exhaustion among FLNMs. By explicitly demonstrating their support, superiors can serve as facilitators, enabling FLNMs to feel acknowledged by hospital management. This acknowledgment may foster better health outcomes among their teams. More broadly, this sense of recognition proves particularly crucial in large bureaucratic organizations, which remain a defining feature of contemporary healthcare systems [81].

### 5.2. Implications for Nursing Management

Given the connection between psychological stress and emotional exhaustion in workplaces, it is imperative to factor in the workplace environment when developing strategies to mitigate these issues. It may be beneficial to conduct periodic assessments of FLNMs' perceptions through means such as employee *pulse* surveys. To address FLNMs' working environment, it is essential to ascertain their perspectives on various aspects, including workload, workplace dynamics, and their experiences with hierarchical support. This approach can facilitate the identification of resources that would enable FLNMs to fulfill their responsibilities more effectively. By conducting these surveys on a regular basis, healthcare organizations can enhance their responsiveness in providing the necessary resources to support FLNMs.

Our findings could also prompt further inquiry into stress resilience among healthcare workers in managerial roles. By disclosing their stresses, FLNMs run the risk of being stigmatized and labeled as incompetent or weak. In practice, the subject of stress remains taboo, with its causes and manifestations being internalized [82]. It would be worthwhile to provide FLNMs with psychologically secure spaces and opportunities to express their concerns and feel heard.

According to LMX theory, a low level of leader loyalty may indicate that the relationship between the superior and their subordinate is still developing and not yet fully mature [46]. Our study's findings indicate that healthcare organizations should consider implementing strategies to foster or reinforce the relationships between FLNMs and their superiors by creating events where they can meet and discuss their respective practices. It is suggested that FLNMs should play a more active role in the decision-making process. Their superiors could seek their opinions and perspectives more often when a project needs to be implemented within the hospital. Their superiors could then function as spokespersons to the board members of the hospital. For instance, FLNMs' superiors could publicly support FLNMs with management when FLNMs encounter difficulties in managing absenteeism within their unit or teams. In this context, they could highlight the measures that have already been implemented by the FLNMs to address this issue, which may serve as a catalyst for requests for staff replacements and/or understaffing related issues. Likewise, it would be advisable for FLNMs and their superiors to convene for regular meetings. The focus of these meetings could be on the FLNMs' perceptions of their roles and the demands of their work. The development of effective communication skills and the nurturing of close relationships can facilitate the timely identification and management of stress, thereby preventing its escalation into emotional exhaustion. This individual meeting is more personal in nature; by providing assistance to the FLNMs, their superiors create a climate of trust, which is a necessary component for the development of a high-quality LMX relationship.

Healthcare organizations must recognize the long-term negative consequences of presenteeism, which not only undermines FLNMs' health but also generates additional financial costs. Promoting a culture that encourages FLNMs to stay home when ill supports recovery and prevents further strain. Presenteeism can serve as an indicator of elevated stress, often linked to excessive workload or interpersonal conflicts. Identifying it as such may prompt supervisors to investigate underlying issues. Furthermore, presenteeism among FLNMs may influence their nursing staff through social learning, reinforcing this behavior within teams.

### 5.3. Limitations and Future Research Directions

While this study reports interesting features, it also has some limitations. First, its cross-sectional nature reduces the possibility of establishing causal relationships between the various variables examined. Future research would benefit from examining the temporal order and causal direction between the variables studied (e.g., reciprocal or reverse effects) using more robust research designs (e.g., prospective or longitudinal designs). Likewise, this study provides insights into an underlying mechanism that contributes to emotional exhaustion among FLNMs: presenteeism. This is an emerging issue, and it would be relevant to continue researching this phenomenon and examining its impact on FLNMs' health. Given the present study's focus on the French hospital sector, further research extending to other European countries would be highly valuable, ideally culminating in a comparative analysis. A notable constraint of the present study is the modest sample size. Consequently, subsequent investigations should consider employing larger samples to enhance the robustness and generalizability of the findings, both at national and international levels.

## 6. Conclusion

This study is relevant as it examined an understudied population: FLNMs. Based on both COR and LMX theories, it shows that psychological stress correlates positively with FLNMs' emotional exhaustion and presenteeism (measured in terms of productivity losses). This study provides a more comprehensive understanding of the process that leads to emotional exhaustion by showing presenteeism's mediating effect. Given the buffering effect of leader loyalty, this study also suggests that healthcare organizations and their managers should pay more attention to enhancing the relationship between FLNMs and their superiors. The results encourage further research (qualitative or quantitative ones) on FLNMs. Indeed, emotional exhaustion is one of the core dimensions of burnout, which is why it should be taken seriously. Such research could facilitate a more profound understanding of the working conditions of these professionals and encourage hospital organizations to prioritize the welfare of their management staff.

## Figures and Tables

**Table 1:** Descriptive statistics and intercorrelations.

Variables	*M*	SD	1	2	3	4
1. Psychological stress	2.92	0.60	0.77			
2. Presenteeism	3.21	0.71	0.344^∗∗^	0.62		
3. Emotional exhaustion	—	—	0.213^∗∗^	0.083	—	
4. Leader loyalty	3.35	1.06	−0.191^∗^	−0.084	−0.161^∗^	0.90

*Note:* Bold values represent Cronbach's alphas. Emotional exhaustion was measured with a single item. *N* (listwise) = 161. *M* = mean.

Abbreviation: SD, standard deviation.

^∗^ *p* < 0.05.

^∗∗^ *p* < 0.01.

**Table 2:** Results of confirmatory factor analysis.

Models	*χ* ^2^	df	*χ* ^2^/df	CFI	TLI	RMSEA	SRMR
1-factor model	203.434	27	7.535	0.655	0.540	0.201	0.152
2-factor model (psychological stress and LL together)	145.931	26	5.61	0.765	0.675	0.169	0.135
2-factor model (presenteeism and LL together)	123.593	26	4.75	0.809	0.736	0.153	0.125
2-factor model (psychological stress and presenteeism together)	76.121	26	2.93	0.902	0.864	0.109	0.068
3-factor model	40.407	24	1.68	0.968	0.952	0.065	0.052

*Note: N* = 161.

Abbreviations: CFI, Comparative Fit Index; df, degree of freedom; LL, leader loyalty; RMSEA, root mean square error of approximation; SRMR, standardized root mean square residual; TLI, Tucker–Lewis Index.

**Table 3:** Results of mediated moderation analysis (Part 1).

Variables	Β	SE	*T*	*p*	LBCI (95%)	UBCI (95%)
*DV = Presenteeism | R2 = 15.13% | F = 9.3305 (p* < 0.001)						
Constant	2.0276	0.3459	5.8615	0.0000	1.3443	2.7108
Gender	−0.2547	0.1384	−1.8401	0676	−0.5281	0.0187
Age	0.0618	0.0345	1.7916	0.0751	−0.0063	0.1299
Psychological stress	0.3912	0.0865	4.5215	0.0000	0.2203	0.5621

*DV = Emotional exhaustion | R2 = 10.66 | F = 3.0624 (p* < 0.01)						
Constant	1.5549	0.8686	1.7901	0.0754	−0.1610	3.2708
Gender	0.0274	0.1346	0.2036	0.8390	−0.2385	0.2934
Age	−0.0172	0.0339	−0.5086	0.6118	−0.0841	0.0497
Psychological stress	0.1945	0.0891	2.1817	0.0306	0.0184	0.3706
Presenteeism	0.6215	0.2367	2.6256	0.0095	0.1539	1.0892
Leader loyalty	0.4863	0.2170	2.2414	0.0264	0.0577	0.9149
Presenteeism × leader loyalty	−0.1722	0.0642	−2.6839	0.0081	−0.2990	−0.0455

*Note: N* = 161. *Bootstrap* analyses are based on 5000 replications (corrected and accelerated bias).

Abbreviations: DV, dependent variable; LBCI, lower bound confidence interval; SE, standard error; UB, upper bound confidence interval.

**Table 4:** Results of mediated moderation analysis (Part 2).

Variables	Effect	SE	*t*	*p*	LBCI (95%)	UBCI (95%)
*Direct effect of psychological stress on emotional exhaustion*						
Psychological stress	0.1945	0.0891	2.1817	0.0306	0.0184	0.3706

*Indirect effect: psychological stress ⟶ presenteeism ⟶ emotional exhaustion*						
Percentiles	Leader loyalty (moderator)		Effect	Boot SE	Boot LBCI	BOOT UBCI
16th	2.0000		0.1084	0.0627	0.0035	0.2532
50th	3.6667		−0.0039	0.0278	−0.0565	0.0545
84th	4.3333		−0.0488	0.0379	−0.1291	0.0220

*Index of mediated moderation (MEDMOD)*						
			Index	Boot SE	Boot LBCI	BOOT UBCI
Leader loyalty (moderator)			−0.0674	0.0352	−0.1471	−0.0076

*Note: N* = 161. *Bootstrap* analyses are based on 5000 replications (corrected and accelerated bias).

Abbreviations: Boot LBCI, *bootstrap* lower bound confidence interval; Boot SE, *bootstrap *standard error; Boot UBCI, *bootstrap* upper bound confidence interval; DV, dependent variable; LBCI, lower bound confidence interval; SE, standard error; UB, upper bound confidence interval.

## Data Availability

The data that support the findings of this study are available from the corresponding author upon reasonable request.
